# Differential Signalling and Kinetics of Neutrophil Extracellular Trap Release Revealed by Quantitative Live Imaging

**DOI:** 10.1038/s41598-017-06901-w

**Published:** 2017-07-26

**Authors:** Maarten van der Linden, Geertje H. A. Westerlaken, Michiel van der Vlist, Joris van Montfrans, Linde Meyaard

**Affiliations:** 1 Laboratory of Translational Immunology, Department of Immunology, University Medical Centre Utrecht, Utrecht, The Netherlands; 2 Department of Paediatric Immunology and Infectious Diseases, University Medical Centre Utrecht, Utrecht, The Netherlands

## Abstract

A wide variety of microbial and inflammatory factors induce DNA release from neutrophils as neutrophil extracellular traps (NETs). Consensus on the kinetics and mechanism of NET release has been hindered by the lack of distinctive methods to specifically quantify NET release in time. Here, we validate and refine a semi-automatic live imaging approach for quantification of NET release. Importantly, our approach is able to correct for neutrophil input and distinguishes NET release from neutrophil death by other means, aspects that are lacking in many NET quantification methods. Real time visualization shows that opsonized *S. aureus* rapidly induces cell death by toxins, while actual NET formation occurs after 90 minutes, similar to the kinetics of NET release by immune complexes and PMA. Inhibition of SYK, PI3K and mTORC2 attenuates NET release upon challenge with physiological stimuli but not with PMA. In contrast, neutrophils from chronic granulomatous disease patients show decreased NET release only in response to PMA. With this refined method, we conclude that NET release in primary human neutrophils is dependent on the SYK-PI3K-mTORC2 pathway and that PMA stimulation should be regarded as mechanistically distinct from NET formation induced by natural triggers.

## Introduction

The discovery of neutrophil extracellular traps (NETs) has enriched our knowledge on the anti-microbial strategies that neutrophils use to fight invading pathogens. NETs are fibres of decondensed chromatin decorated with granular proteins and are released in the extracellular milieu to kill various pathogens^1–4^. Opposite to the beneficial role of NETs to innate immune defence, NET formation has been shown to contribute to inflammation in non-infectious diseases. The formation of NETs in tissue or inside the vasculature could lead to clot forming, metastasis or exposure of autoantigens and thus contribute to the pathogenesis of thrombosis^5^, cancer^6^ and autoimmune inflammatory diseases^7–10^, respectively. In systemic lupus erythematosus (SLE), the presence of LL-37, human neutrophil peptide (HNP) and autoantibodies against these specific cellular components supports the formation of immune complexes (ICs), which trigger neutrophils to release NETs^11^. In addition, monosodium urate (MSU) crystals present in joints of gout patients have been described to abundantly induce NET release^12^.

Many of the current reports on NET release are based on *in vitro* studies using the non-physiological stimulus phorbol myristate acetate (PMA). PMA is a potent stimulator of protein kinase C (PKC), which in turn activates the nicotinamide adenine dinucleotide phosphate (NADPH)-oxidase complex leading to abundant generation of reactive oxygen species (ROS)^13^. Although PMA has been used to study the biology of NETs in many publications, it is not a physiological stimulus and thus will differ from NET release induced by microbial and endogenous stimuli.

To experimentally address the release of NETs *in vitro*, in recent years many strategies have been developed. Microscopic techniques are most often used to determine and quantify NETs (reviewed by^14^). Microscopy is useful to visualize NET release and to detect specific NET components, but has its limitations. Non-automatic quantification of fixed time point microscopic experiments is rather inaccurate, because the results could easily be biased by the observer and it is impossible to correct for neutrophil input. Another technique combines microscopy with flow cytometry, also called multispectral imaging flow cytometry (MIFC), and quantifies NET release by visualization and side-scatter and is able to identify NET components by multiple fluorescence staining^15^. MIFC allows unbiased quantitative analysis, however it focuses on neutrophils in an early phase of NET release and may miss neutrophils that actually release NETs.

An important limitation that all these visualization techniques have in common is that they lack the possibility to study NET kinetics, on which there is currently no consensus. Rapid NET release has been shown in response to *Staphylococcus aureus* (*S. aureus*)^16^, Fc receptor (FcR) ﻿activation﻿^17, 18^, MSU^19^, *Candida albicans* (*C. albicans*) hyphea^20^, and *Leishmania* ^21^, while PMA^18^, ICs^22^ and *C. albicans* yeast^20^ trigger NET formation at later time points. Many of these studies were performed with fluorescence plate reader experiments using DNA binding dye’s (i.e. PicoGreen or Sytox Green), however this will not distinguish DNA expelled as NETs from other forms of DNA release.

Live imaging approaches were shown to enable following single neutrophils and visualize NET release, which makes it the technique of choice to study neutrophil morphology and kinetics^23, 24^. Recently, a novel semi-automated NET quantification has been described^25^. This technique is able to detect PMA-induced NETs based on the surface of Sytox Green staining. Because this procedure does not require extensive processing of cells, it has been shown to be reproducible and valid compared to manual counting. Here, we have used live imaging and a validated semi-automatic approach to quantify NET release in response to physiological stimuli in healthy donors (HDs) and in patients with chronic granulomatous disease (CGD). This allowed us to obtain novel information on the kinetics and underlying signalling pathways that result in NET release following non-inflammatory and inflammatory stimuli and it indicates that PMA is not a good model for any of those.

## Results

### A semi-automatic quantification approach to analyse live imaging NET release

We performed a live imaging assay to monitor NET release over time that uses Hoechst stain at time point zero to control for neutrophil input and validated an earlier described analysis approach that specifically quantifies NET release^25^. To discriminate NETs from dying, Sytox Green permeable neutrophils (further referred as Sytox Green+ neutrophils), we assessed maximal surface of Sytox Green+ neutrophils and the minimal size of NETs. Using this approach we set thresholds for noise (35 µm^2^), Sytox Green+ neutrophils (35–68 µm^2^) and NETs (>68 µm^2^). The use of Fiji macros ensured a semi-automatic quantification approach (Fig. S1).

**Figure 1:**
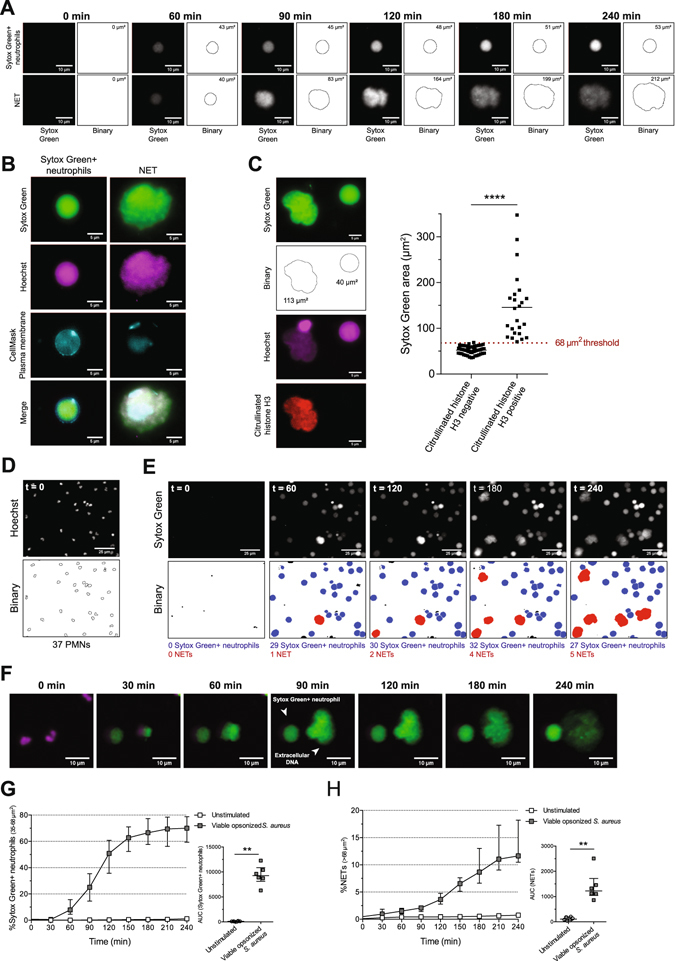
Surface-based analysing approach to quantify NET release. Neutrophils from HDs were stimulated with viable opsonized *S. aureus* and monitored over time for 4 hours using Hoechst 33342 and Sytox Green. (**A**) Sytox Green images from live imaging were transferred to binary images and the size of Sytox Green+ neutrophils (<53 µm^2^) and NETs (83–212 µm^2^) were measured. (**B**) Fluorescence microscopy using CellMask plasma membrane dye revealed that the DNA of Sytox Green+ neutrophils is intracellular and NETs are extracellular. (**C**) Citrullinated histone H3 is only present in Sytox Green particles with a surface above 68 µm^2^, which confirms that these are NETs. Data from 3 independent experiments are presented. Statistical significance (****P < 0.0001) was determined by two-tailed Mann-Whitney test. For quantification, the amount of neutrophils in t = 0 (**D**), Sytox Green+ neutrophils (blue), and NETs (red) in multiple time points (**E**) were determined. (**F**) Indicated time points of live imaging (Supplemental video 1; Hoechst 33342 in magenta and Sytox Green in green) showed the binding of Sytox Green to intracellular DNA (Sytox Green+ neutrophil) and extracellular DNA. For quantification, the amount of Sytox Green+ neutrophils or NETs were divided by the amount of neutrophils in t = 0 and expressed as percentage of Sytox Green+ neutrophils (**G**) or NETs (**H**). Data from 5 independent experiments are presented as median ± interquartile range. Statistical significance (**P < 0.01) was determined by two-tailed Mann-Whitney test. The images in **A–F** are representative of at least 3 experiments with neutrophils from different donors.

To quantify NETs, the Sytox Green images were transferred to binary images and the size of Sytox Green+ neutrophils as well as the NETs was determined. Sytox Green+ neutrophils typically covered a surface of between 40 and 55 µm^2^ while NETs covered a surface of over 68 µm^2^ (Fig. 1A). In addition, NET surface increased over time because of diffusion while the size of Sytox Green+ neutrophils remained below 68 µm^2^.

We used three separate approaches to confirm the differences between Sytox Green+ neutrophils and NETs. First, we counted all Sytox Green particles at time point 60 min and 240 min after stimulation with viable opsonized *S. aureus*. After 60 min, the majority of Sytox Green particles covered a surface between 0 and 40 µm^2^ but remained below 68 µm^2^. The surface of these Sytox Green particles increased (20–50 µm^2^) after 240 min of stimulation. Moreover, NETs with a surface above 68 µm^2^ appeared after 240 min of stimulation (Fig. S2). Second, with CellMask plasma membrane dye we secured that Sytox Green staining of Sytox Green+ neutrophils is intracellular while NETs are extracellular (Fig. 1B). Finally, citrullinated histone H3 was present only in the extracellular DNA while the Sytox Green+ neutrophil lacks citrullinated histone H3 (Fig. 1C). This confirms that the DNA was expelled in the form of NETs and that our NET surface-based threshold of >68 µm^2^ is valid to genuine NETs.

We determined the number of neutrophils over time and showed similar amount of neutrophils at time point zero (t = 0) and 240 min (Fig. S3). Subsequently, we used t = 0 for correcting neutrophil input. Hoechst images were transferred to binary images and the number of neutrophils was determined (Fig. 1D). Likewise, with Sytox Green images at multiple time points, the number of Sytox Green+ neutrophils (in blue) and NETs (in red) was determined (Fig. 1E).

Upon exposure to viable opsonized *S. aureus*, permeabilization of the neutrophil plasma membrane occurred within 30 to 60 min where after Sytox Green entered the neutrophils. After 90 min, the neutrophil plasma membrane of part of the cells broke and DNA was released in the extracellular environment, while part of the cells stayed Sytox Green positive but did not release NETs (Fig. 1F and Supplemental video 1).

Quantification demonstrated that within the first 60 min of exposure to viable opsonized *S. aureus*, neutrophils became Sytox Green+ with ~70% being positive after 240 min (Fig. 1G). The release of NETs occurred after 90 min and ~12% of cells released NETs after 240 min of stimulation with viable opsonized *S. aureus* (Fig. 1H). Taken together, these data show that we have validated a surface-based semi-automatic analysing approach which is able to specifically quantify physiologically-induced NET release over time and correct for cellular input.

### Live imaging distinguishes NET release from toxin-induced cell death

**Figure 2:**
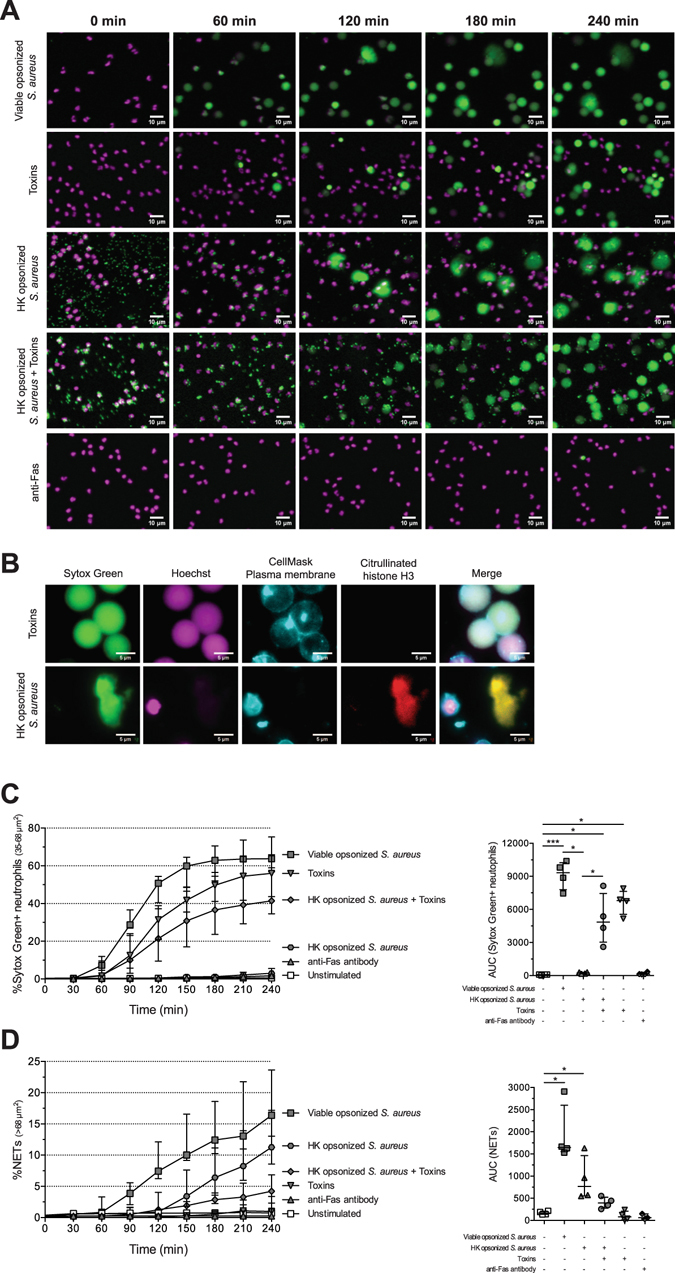
Distinguishing toxin-induced cell death and apoptosis from NET release. Neutrophils from HDs were stimulated with anti-Fas antibody, toxins from overnight culture of *S. aureus* or viable and HK opsonized *S. aureus* and monitored over time for 4 hours. (**A**) Indicated time points from live imaging (Supplemental video 3) showed that neutrophils release NETs in response to HK opsonized *S. aureus* while stimulation with toxins alone or a combination of toxins and HK opsonized *S. aureus* mainly resulted in Sytox Green+ neutrophils. Anti-Fas antibody neither induced Sytox Green+ neutrophils nor NET release. (**B**) Fluorescence microscopy using CellMask plasma membrane dye and anti-citrullinated histone H3 antibody confirms the presence of extracellular DNA in the form of NETs. Quantification of Sytox Green+ neutrophils (**C**) and NETs (**D**) of 2 independent experiments performed with 4 donors. The data are presented as median ± interquartile range. Statistical significance (*P < 0.05; ***P < 0.001) was determined by Kruskal-Wallis test. The images in **A** and **B** are representative of at least 3 experiments with neutrophils from different donors.

Upon exposure to viable opsonized *S. aureus*, neutrophils either released NETs or only became permeable, becoming Sytox Green+ without expulsion of their DNA into extracellular space. Even after prolonged incubation, the Sytox Green+ neutrophils did not release NETs (Fig. S4 and Supplemental video 2), suggesting exposure to viable opsonized *S. aureus* results in two separate mechanisms of cell death. Since *S. aureus* toxins are known to induce cell death^24^, we exposed neutrophils to toxins obtained from an overnight culture of *S. aureus* and monitored neutrophil fate over time. After exposure with toxins, neutrophils became permeable and Sytox Green+ (Fig. 2A). The lack of citrullinated histone H3 confirms that toxin-induced Sytox Green+ neutrophils are not NETs (Fig. 2B).

Our time-lapse NET quantification revealed that the first Sytox Green+ cells appeared after 40 minutes and ~58% of the cells being Sytox Green+ after 240 min without the release of NETs (Fig. 2C,D). Interestingly, not a single neutrophil became Sytox Green+ without also releasing citrullinated histone H3 positive NETs after stimulation with heat-killed (HK) opsonized *S. aureus*, which cannot produce toxins (Fig. 2B,C,D and Supplemental video 3). To demonstrate the difference between HK and live opsonized *S. aureus*-induced NET release is because of toxin production, we supplemented HK opsonized *S. aureus* with toxins. The neutrophil response with HK opsonized *S. aureus* with exogenous toxins mimicked that of live opsonized *S. aureus*: neutrophils became Sytox Green+, but the majority of these cells did not produce NETs.

Thus, bacterial toxins are responsible for the permeabilization of the neutrophil plasma membrane after which Sytox Green enters the neutrophil. Our live imaging approach is able to distinguish NET release from toxin-induced cell death even when both processes are occurring in the same sample. Furthermore, upon stimulation with anti-Fas antibody to induce apoptosis, no Sytox Green+ neutrophils n or NETs were present (Fig. 2A,C,D). This shows that we have developed an assay and method of analysis that distinguishes NETs from other cell death mechanisms.

### MSU induces rapid NET release

**Figure 3:**
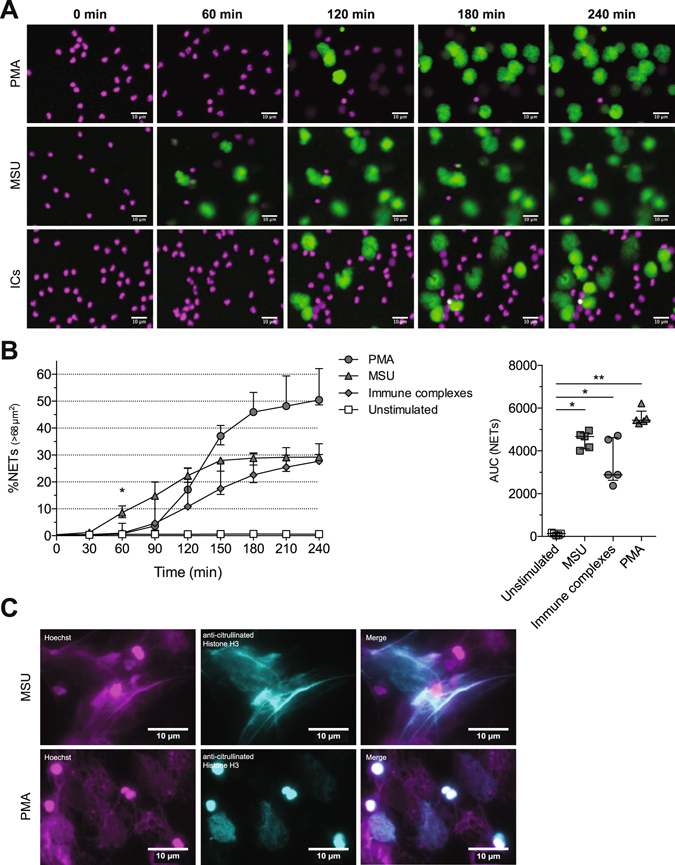
MSU induces rapid NET release. Neutrophils from HDs were stimulated with MSU, ICs and PMA and monitored over time for 4 hours. (**A**) Indicated time points from live imaging showed MSU-induced NET release within 60 min while NET release in response to ICs and PMA occurred within 120 min. (**B**) Quantification of NETs of 5 independent experiments. The data are presented as median ± interquartile range and statistical significance (*P < 0.05; **P < 0.01) was determined by Kruskal-Wallis test. (**C**) Fluorescence microscopic experiments revealed the presence of citrullinated histone H3 in the extracellular DNA after 180 min stimulation with MSU and PMA. The images in **A** and **C** are representative of at least 4 experiments with neutrophils from different donors.

Also in non-infectious conditions, neutrophils can be triggered by host factors to release NETs. The exposure of neutrophils to MSU or ICs results in abundant NET release^22, 26^. PMA is a non-physiological stimulus often used for study of NETs *in vitro* ^24^. Upon stimulation with MSU, ICs or PMA, neutrophils released NETs (Fig. 3A). NET release in response to ICs and PMA started around 90 min while MSU-induced NET release occurred already within 60 min, starting as soon as 30 min (Fig. 3B). The presence of citrullinated histone H3 in the extracellular DNA of neutrophils stimulated with PMA and MSU confirmed that the DNA was expelled in the form of NETs (Fig. 3C). This shows that NET kinetics is dependent on the type of stimulus, with MSU inducing rapid NET release.

### PMA and physiological stimuli activate different pathways to induce NETs

To investigate the underlying pathway of NET release, we used chemical inhibitors to target specific downstream signalling molecules. Neutrophils were pre-treated with various inhibitors before exposure to live opsonized *S. aureus*, MSU, and PMA. We first determined the optimal concentration of the chemical inhibitors and chose the highest concentrations that did not induce Sytox Green signal (dying cells) in otherwise non-stimulated neutrophils (Fig. S5A).

**Figure 4:**
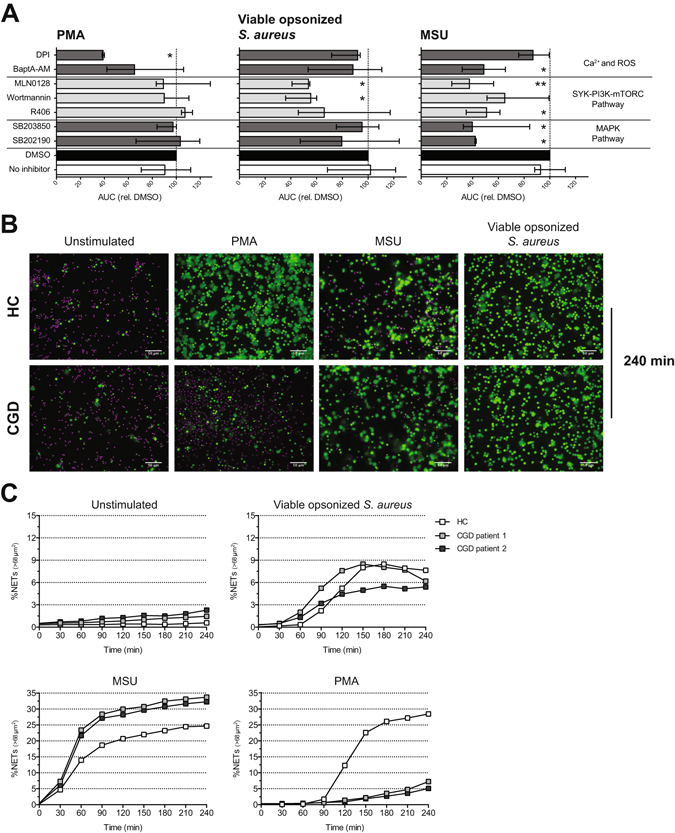
PI3K, SYK and mTORC2 are important players of the underlying signalling pathway of NET release. Neutrophils from HDs were incubated with Wortmannin (PI3K inhibitor), R406 (SYK inhibitor), SB203580 and SB202190 (p38 MAPK inhibitors), MLN0128 (pan-mTORC inhibitor), BAPTA-AM (Chelator of Ca^2+^), and DPI (NADPH oxidase inhibitor) before stimulation with MSU, PMA and viable opsonized *S. aureus*. (**A**) AUC of NET quantification of 3 independent experiments are presented as median ± interquartile range and statistical significance (*P < 0.05; **P < 0.01) was determined by Kruskal-Wallis test. Neutrophils from one HC and two CGD patients were stimulated with MSU, PMA and opsonized *S. aureus* and monitored over time for 4 hours. (**B**) Merge images (Hoechst 33342 in magenta and Sytox Green in green) of time point 240 min showed decreased NET release of CGD neutrophils in response to PMA compared to HC neutrophils. (**C**) Quantification of NET release of 1 experiment with 1 HC and 2 CGD donors.

The release of NETs in response to PMA was inhibited with 40–60% in the presence of DPI (NADPH oxidase inhibitor) and BAPTA-AM (chelator of Ca^2+^) while treatment of neutrophils with Wortmannin (PI3K inhibitor), R406 (SYK inhibitor), MLN0128 (pan-mTORC inhibitor), SB203580 and SB202190 (p38 MAPK inhibitors) did not affect NET release in response to PMA (Fig. 4A). To further study the role of NADPH oxidase in NET release, we used neutrophils from two male CGD patients (twins). These CGD patients contain a homozygous delta GT mutation in the *NCF1* gene (gene encoding the p47phox protein; a component of NADPH oxidase), which leads to low levels of ROS production. PMA-induced NET release was abrogated in CGD neutrophils compared to neutrophils from a HD (Fig. 4B,C).

NET release induced by MSU and viable opsonized *S. aureus* was reduced with 40–60% when neutrophils were treated with Wortmannin, R406 and MLN0128 (Fig. 4A). Treatment of neutrophils with Rapamycin (mTORC1 inhibitor) did not attenuate NET release in response to these physiological stimuli (Fig. S5B). In addition, Celastrol (NF-κB inhibitor), Bay-11-7082 (IκB phosphorylation inhibitor), GW5074 (cRaf1 kinase inhibitor), U-73122 (PLC inhibitor), Bafilomycin A (vacuolar-type H+ -ATPase inhibitor), JNKi (JNK inhibitor) or Mytoquinone (mitochondrial ROS inhibitor) did not affect NET release (Fig. S5C). This suggests the importance of the SYK-PI3K-mTORC2 signalling pathway in NET release triggered by MSU and viable opsonized *S. aureus*, since the pan-mTORC inhibitor attenuated NET release and the mTORC1 inhibitor did not.

To prove a distinct underlying signalling mechanism in PMA-induced NET release compared to NET formation induced by MSU and viable opsonized *S. aureus*, we again used DPI treated neutrophils or neutrophils from CGD patients. Upon stimulation with MSU and viable opsonized *S. aureus*, DPI-treated neutrophils as well as CGD neutrophils did release NETs (Fig. 4A,B). Quantification revealed that NET release of CGD neutrophils is comparable or even increased compared to NET release from a HD (Fig. 4C). Although we only used one HD in this experiment, the release of NETs in response to PMA, live opsonized *S. aureus* and MSU of this HD is comparable with other HDs from previous experiments. Together, our data indicate that calcium and NADPH oxidase-induced ROS play an important role in PMA-induced NET release. In contrast, NET release in response to MSU and viable opsonized *S. aureus* acts via the SYK-PI3K-mTORC2 pathway and is independent of NADPH oxidase-mediated ROS.

## Discussion

NETs serve as an innate immune defence mechanism to protect the host against invading pathogens, while on the other hand uncontrolled NET release can contribute to disease development and -progression. To visualize and quantify NET release *in vitro*, many experimental techniques are described, including fixed time point microscopy^27^, live imaging^23, 24, 28, 29^, image-based flow cytometry^15^, fluorescence plate reader assay using DNA binding dye’s^16, 30, 31^ and ELISA based assays^30^. These techniques are useful for studying NET components, morphology and kinetics. However, it is important to be aware of the limitations of each of these techniques. In this study, we use a semi-automatic approach to quantify NET release in a live imaging assay, which is based on previous described methods^25, 32^. Our approach was able to correct for neutrophil input and distinguishes NET release from other mechanisms of cell death, crucial aspects that are lacking in many NET quantification methods.

A unique mechanism of NET release in response to *S. aureus* was previously described: rapid (within minutes) and without breaching the neutrophil plasma membrane^16^. Consistent with this study, we previously have shown that, when using a fluorescence plate reader assay, Sytox Green fluorescence appears within 30 min when neutrophils were exposed to viable *S. aureus* ^16, 19^. We concluded at the time that neutrophils are able to rapidly release NETs in response to these stimuli. However, *S. aureus* secrete pore-forming toxins that contribute to bacterial virulence and are able to permeabilize neutrophils for DNA dyes that are normally cell membrane impermeable^33–35^. We now show that cytolytic toxins from viable *S. aureus* induce rapid permeabilization of the neutrophil plasma membrane allowing for Sytox Green influx and nuclear DNA staining without histone H3 citrullination. In strong contrast, actual NET release occurs at a later time point (Supplemental video 2). Indeed, HK *S. aureus* that cannot secrete toxins, are not able to permeabilize neutrophils and only trigger NET release. Without imaging, i.e. with a fluorescence plate reader, it is not possible to discriminate between nuclear DNA and NETs. Thus, we conclude that imaging is essential for measuring NET release.

Visualization of NET release in a live imaging assay shows that expelled DNA from the neutrophil diffuses around the neutrophil itself^23–25^. In contrast, fixed time point microscopic experiments show DNA structures that seem to be pulled apart and cover a large surface^25, 36^. Similar structures of NETs were shown *in vivo* where blood flow and moving cells result in stretched NETs^37–39^. *In vitro*, this stretched morphology is induced by pipetting (Fig. S6). This shows that quantification of live imaging NET release is more accurate than quantification of NET release visualized with fixed time point microscopy.

Neutrophils rapidly kill pathogens by phagocytosis or by the secretion of proteases to protect the host against severe infection^40, 41^. NETs have also been shown to eliminate microbes^1–4^. So far, it is not known which signal triggers the neutrophil to release their nuclear content in the extracellular milieu. The release of NETs could act as a cell death mechanism in which the neutrophil sacrifices itself to protect others. This suggests that NET release is a late response, which is induced when other anti-microbial strategies have failed. In addition, neutrophils sense microbe size and release NETs in response to large pathogens^42^, arguing for an active process that occurs directly after neutrophil contact with the microbe. In contrast with fluorescence plate reader data from our previous study^19^, we here show that only MSU triggers rapid NET release while NET formation in response to opsonized *S. aureus*, PMA, and ICs occurs at a later phase. Others have also shown that PMA^18^ and ICs^22^ induce late NET release. Although these experiments were performed with a fluorescence plate reader assay and therefore unable to discriminate between bona fide NETs and permeable cells, we now confirm late phase NET release in response to PMA and ICs. Similarly, fluorescence plate reader experiments revealed that FcR activation^17, 18^, *Leishmania* ^21^, and *C. albicans* hyphea^20^ induce rapid NET release while *C. albicans* yeast^20^ triggers NET formation at a later time point. Our current data underline that these kinetics should be interpreted carefully and analysed in an assay like ours, to determine the difference between cellular permeability and actual NET release.

There is no consensus of the requirement of ROS production for NET formation and current data on NET release suggests there are two differential signalling pathways that induce the release of NET. Direct stimulation of PKC, i.e. by PMA, provokes ROS-dependent NET formation, which diminishes in the presence of DPI. Similarly, DPI was published to attenuate NET release upon stimulation with ICs^22, 43^, anti-LL37^44^ and non-opsonized *S. aureus* ^24^. Indeed, CGD neutrophils do not release NETs upon PMA^23, 24, 45–47^ or non-opsonized *S. aureus* ^24^ stimulation. This demonstrates that some stimuli require NADPH-induced ROS to release NETs. On the contrary, we and others showed that DPI does not affect NET release when neutrophils are stimulated with MSU^19^, ICs^48^, uric acid^49^, *Leishmania* ^21^, ionomycin^50^ and opsonized *S. aureus* ^16, 19^. In addition, it was shown that CGD neutrophils are still able to release NETs when exposed to uric acid^49^, which suggests the presence of a NADPH-oxidase independent NET signalling pathway. We now show that CGD neutrophils as well as DPI-treated neutrophils release NETs upon exposure of MSU and viable opsonized *S. aureus* while PMA-induced NET release is low. So far, data obtained with neutrophils from CGD patients confirm that the role of NADPH oxidase-dependent ROS in NET release is stimulus dependent. However, the discrepancies about the role of ROS in NET release in studies that use DPI are challenging to interpret. We propose two distinct explanations: 1) The effect of DPI is concentration dependent and/or non-specific or; 2) inappropriate NET measuring techniques and quantification methods that lack experimental sensitivity are masking the true effect of DPI on NET release. Thus, the use of a live imaging assay that allows visualization and quantification of NET release and discriminates between actual NETs and other cell death mechanisms is essential for future NET studies.

A wide variety of cell surface receptors on neutrophils act via SYK and PI3K to induce neutrophil activation^51, 52^. FcRs, Toll-like receptors (TLRs) and the P2Y6 receptor are amongst them and could be triggered upon exposure with ICs, opsonized *S. aureus* and MSU^53^, respectively. We show that the SYK-PI3K-mTORC2 pathway is involved in NET release upon physiological stimuli but not in PMA-induced NET release, which is consistent with previous data^22, 54^. In addition, PMA provokes NET release that is morphologically different from other NETs (Supplemental video 4). This indicates that PMA-induced NETs should be regarded as mechanistically distinct from NET formation induced by natural triggers. Therefore we recommend to interpret statements from NET studies performed with PMA with care.

This study highlights the potency of a live imaging technique to study NET release. Using a validated semi-automatic quantification approach, we are able to correct for neutrophil input and distinguish between NETs and other cell death mechanisms, two aspects that increase the accuracy of NET quantification. At the same time, we reveal the limitations of fluorescence plate reader assays using cell-impermeable DNA binding dyes for measurement of NET release. The use of PMA to induce NET release should be avoided or interpreted with great care because it induces NET release completely different from all physiological stimuli.

## Materials and Methods

### Isolation of human neutrophils

Peripheral blood from HDs and from two CGD patients (twin boys) was collected in sodium-heparin tubes (Greiner Bio-One). All blood donors gave informed consent. Patients with CGD were recruited in the outpatient clinic of the department of Paediatric Immunology and Infectious Diseases of the UMC Utrecht. Diagnosis of CGD was determined functionally by absence of respiratory burst upon stimulation in neutrophil (Phagoburst, BD Bioscience) and genetically by Sanger sequencing of the *NCF1* gene showing a pathogenic homozygous mutation (delta GT). Neutrophils were isolated by Ficoll-Paque (GE Healthcare) density gradient centrifugation, after which erythrocytes were lysed in ammonium chloride buffer (155 mM NH_4_Cl; 10 mM KHCO_3_; 0.1 mM EDTA in double-distilled H_2_O; pH = 7.2). Cells were resuspended in RPMI 1640 (Life Technologies) supplemented with 10% (v/v) heat-inactivated (HI) foetal bovine serum (FBS) (Biowest) and 50 U/ml Penicillin-Streptomycin (referred to as RPMI 10% hereafter). Purity of isolated neutrophils was analysed using the CELL-DYN Emerald and was >88%. All experiments were performed in accordance with relevant guidelines and regulations approved by the Medical Ethical Committee of the University Medical Centre Utrecht.

### Bacterial culture and preparation

*S. aureus* Wood 46 was grown up to exponential phase at 37 °C under aerobic conditions in Todd-Hewitt Broth (THB) containing 1% (v/w) yeast extract. After quantification of bacteria by measuring OD_600_ (2 × 10^8^ CFU with OD_600_ = 0.4), bacteria were washed twice with chilled PBS and opsonized for 30 min at 37 °C with 10% HI human pooled serum in PBS. In some experiments, bacteria were HK for 60 min at 70 °C before opsonisation.

### Chemical inhibitors

To study the underlying signalling NET pathway, several chemical inhibitors were used. Before stimulation, neutrophils were pre-treated for 30 min at 37 °C in RPMI 10% with DMSO (vehicle control), 1 µM Wortmannin (PI3K inhibitor; Sigma-Aldrich), 1 µM R406 (SYK inhibitor; Selleckchem), 10 µM SB203580 (p38 MAPK inhibitor; Selleckchem), 10 µM SB202190 (p38 MAPK inhibitor; Santa Cruz Biotechnology) 10 µM MLN0128 (pan-mTORC inhibitor; Selleckchem), 10 µM BAPTA-AM (chelator of Ca^2+^;Focus Biomolecules), 1 µM DPI (NADPH oxidase inhibitor; Sigma-Aldrich), 1 µM Rapamycin (mTORC1 inhibitor; Selleckchem), 1 µM Celastrol (NF-κB inhibitor; InvivoGen), 0.1 µM Bay-11-7082 (IκB phosphorylation inhibitor; InvivoGen), 0.1 µM GW5074 (cRaf1 kinase inhibitor; Santa Cruz Biotechnology), 0.1 µM U-73122 hydrate (PLC inhibitor; Sigma-Aldrich), 1 nM Bafilomycin A (vacuolar-type H+ -ATPase inhibitor; Sigma-Aldrich), 0.1 µM JNKi II 420128 (JNK inhibitor; Merck Millipore) or 0.1 µM Mytoquinone (mitochondrial ROS inhibitor; BIOTREND).

### Preparation of ICs

Insoluble ICs were formed by using human serum albumin (HSA) (Sanquin) and rabbit polyclonal anti-HSA IgG (Sigma Aldrich) as described previously^55^. Briefly, a mix of 5 µg HSA and 45 µg rabbit-anti-HSA antibody was made in a final volume of 50 µl PBS. After 1 h incubation at 37 °C, insoluble ICs were formed and used for stimulation of neutrophils.

### Microscopic live imaging NET assay

Neutrophils were incubated in RPMI 10% containing 20 μM Hoechst 33342 for 30 min at 37 °C, in the presence or absence of chemical inhibitors, and washed twice with RPMI 1640 (without phenol red) supplemented with 2% FBS, 50 U/ml Penicillin-Streptomycin, and 10 mM HEPES (referred to as RPMI-pr 2% hereafter). A total of 5 × 10^4^ neutrophils were seeded on 0.001% poly-L-lysine (Sigma Aldrich) pre-coated wells of a clear bottom 96-wells plate (Ibidi) and challenged with 25 ng/ml PMA (Sigma Aldrich), 100 μg/ml MSU, 10 µg/ml ICs, opsonized *S. aureus* at a MOI of 10. All stimuli were resuspended in RPMI-pr 2% containing 4 nM Sytox Green (Life Technologies). NET release was recorded at 37 °C and 5% CO_2_ on the Pathway 855 bioimaging system (BD Biosciences) with a 10x objective during a period of 240 min. Every 30 min, a set of two images (Exc/Em: 350/461 nm (Hoechst) and 504/523 nm (Sytox Green)) was taken with an Orca high-resolution CCD camera and four fields of view (each 415 × 317 μm) per condition were captured. The system was controlled by the AttoVision software (version 1.7/855). To make movies, images were taken every 2 min.

### Semi-automatic NET quantification approach

This NET quantification approach is based previously described methods^25, 32^. For quantification of NETs, images were processed with Fiji software (version 2.0.0-rc-43/1.51d). Image stacks were created and converted in 8-bit greyscale. To count neutrophils, thresholding of t = 0 Hoechst image was done with “Moments” logarithm followed by watershed segmentation to separate particles that touch. To count NETs, thresholding of Sytox Green stacks were performed with “Li” logarithm followed by watershed segmentation. The amount of Sytox Green+ neutrophils (35–68 μm^2^) or NETs (>68 μm^2^) were divided by the total number of neutrophils in t = 0 to calculated percentages. For semi-automatic quantification analysis, three macros were created for: 1) making stacks, 2) counting neutrophils, and 3) counting Sytox Green+ neutrophils and NETs (Fig. S1).

### Induction of neutrophil apoptosis and toxin-mediated cell death

Neutrophil apoptosis and toxin-mediated cell death was induced as described before^24^. Briefly, neutrophils were incubated with 20 ng/ml anti-human CD95 (Fas) antibody clone EOS9.1 (BioLegend) to induce apoptosis and 25 µg/ml toxins from *S. aureus* to induce cell death. Toxins were obtained by growing *S. aureus* Wood 46 overnight in THB containing 1% yeast. Bacteria were pelleted, supernatant was collected and sterilized with a 0.20 µm filter. Protein concentration was determined with BCA Protein Assay (Thermo Fisher Scientific).

### Immunofluorescence staining of citrullinated histone H3

Neutrophils were resuspended in RPMI-pr 2% and a total of 5 × 10^5^ neutrophils were seeded on 0.001% poly-L-lysine coated glass coverslips. Neutrophils were challenged as described before for 180 min at 37 °C and 5% CO_2_. Medium was gently discarded and neutrophils were fixed with 2% paraformaldehyde (Electron Microscopy Sciences) for 15 min at room temperature (RT). After blocking with 20% normal goat serum (Cell Signaling Technologies) in PBS containing 2% bovine serum albumin (BSA) (Roche) for 30 min at RT, 0.25 µg/ml rabbit-anti-citrullinated histone H3 antibody (Abcam) in PBS/2% BSA was used for incubation overnight at 4 °C. Next day, neutrophils were incubated with 2.5 µg/ml goat-anti-rabbit antibody conjugated with Alexa Fluor 594 (Life Technologies) for 120 min at RT, followed by 30 min of incubation at RT with 5 µM Hoechst 33342 in PBS to stain neutrophil nucleus. Coverslips were mounted in Fluoromount-G (Southern Biotech) and analysed with Pathway 855 bioimaging system with a 20x objective.

For immunofluorescence staining of citrullinated histone H3 in the live imaging NET assay, neutrophils were incubated in RPMI-pr 2% containing Hoechst 33342 and 1x CellMask^TM^ Deep Red Plasma membrane stain (Thermo Fisher Scientific) for 30 min at 37 °C. After washing with RPMI-pr 2%, neutrophils were seeded and challenged with toxins, viable and HK opsonized *S. aureus*. All stimuli were resuspended in RPMI-pr 2% containing Sytox Green. After 180 min incubation at 37 °C and 5% CO_2_, 2 µg/ml rabbit-anti-citrullinated histone H3 antibody was added and incubated for 30 min. A mix of 0.67 µg/ml donkey-anti-rabbit (Life Technologies) and 0.67 µg/ml goat-anti-rabbit antibody, both conjugated with Alexa Fluor 594, was added and incubated for 15 min before NET release was recorded with a 40x objective. Sets of four images (Hoechst, Sytox Green, Exc/Em: 590/617 nm (AF594) and 649/666 nm (CellMask Deep Red)) were taken.

### Statistical analysis

Data were analysed in GraphPad Prism 6. The comparison of two samples was performed by two-tailed Mann-Whitney test and multiple comparisons were established by Kruskal-Wallis test. Each experiment was performed at least three times on independent occasions. *P* < 0.05 was considered significant.

## Electronic supplementary material

Video S1. NET release in response to live opsonized S. aureus.

Video S2. Visualization is necessary to distinguish NET release from toxin-induced cell death.

Video S3. Toxin-induced cell death vs NET release.

Video S4. NET release in response to PMA.

Supplementary data
