# Supplementary material for: Differential Signalling and Kinetics of Neutrophil Extracellular Trap Release Revealed by Quantitative Live Imaging
Source: Sci Rep. 2017 Jul 26;7:6529. doi: 10.1038/s41598-017-06901-w (PMC5529471; doi:10.1038/s41598-017-06901-w)
Supplement: Supplementary file 5 — Supplementary data [file 41598_2017_6901_MOESM5_ESM.pdf]

Differential Signalling and Kinetics of Neutrophil Extracellular Trap Release Revealed by Quantitative Live Imaging

Maarten van der Linden[1], Geertje H.A. Westerlaken[1], Michiel van der Vlist[1], Joris van Montfrans[2] & Linde Meyaard[1]*

Video S1. NET release in response to live opsonized *S. aureus*. Neutrophils from HDs were stimulated with live opsonized *S. aureus* and monitored over time for 4 hours using Hoechst (magenta) and Sytox Green (green). Live imaging showed the binding of Sytox Green to intracellular DNA (Sytox Green+ neutrophil) and extracellular DNA (NET).

Video S2. Visualization is necessary to distinguish NET release from toxin-induced cell death. Neutrophils from HDs were stimulated with viable opsonized *S. aureus* or toxins from *S. aureus* and monitored over time for 4 hours using Hoechst (magenta), Sytox Green (green) and transmitted light. Live imaging showed that NET release occurs in multiple steps. When neutrophils are exposed to viable opsonized *S. aureus*, neutrophils become activated and the nuclear material is spread throughout the neutrophil but remains intracellular. At the moment the plasma membrane breaks, the DNA was released in the extracellular environment. When the neutrophils are exposed to only toxins from *S. aureus*, the cell death that occurs does resembles the first stage of NET release but does not result in actual release of DNA in the extracellular milieu.

Video S3. Toxin-induced cell death vs NET release. Neutrophils from HDs were stimulated with toxins from overnight culture of *S. aureus* or HK opsonized *S. aureus* and monitored over time for 4 hours using Hoechst and Sytox Green. Live imaging showed that neutrophils release NETs in response to HK opsonized *S. aureus* while stimulation with toxins alone resulted in Sytox Green+ neutrophils.

Video S4. NET release in response to PMA. Neutrophils from HDs were stimulated with PMA and monitored over time for 4 hours using Hoechst and Sytox Green.

Figure S1.

Macro	Step by step procedure
1. Creating image sequences	- Open image sequence via import (image sequence)<br>- Save image sequences as Tiff file
2. Counting neutrophils	- Set scale<br>- Set auto Threshold (Moments)<br>- Seperate particles via watershed function<br>- Analyze particles (Size in μm^2: 0-infinity; Circularity is 0-1)
3. Counting Sytox Green+ neutrophils and NETs	- Set scale<br>- Set auto Threshold (Li)<br>- Seperate particles via watershed function<br>- Analyze particles<br>* Sytox Green+ neutrophils (Size in μm^2: 35-68; Circularity is 0-1)<br>* NETs (Size in μm^2: 68-infinity; Circularity is 0-1)

Figure S2.

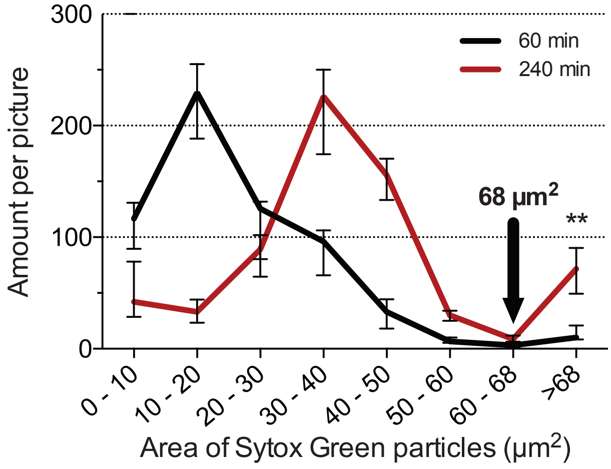

Figure S2. NETs cover a surface above 68 μm^2. Neutrophils from healthy donors were stimulated with opsonized *S. aureus*. On time point 60 min and 240 min after stimulation, Sytox Green surface of all particles per image were determined and the amount of particular Sytox Green particles was defined. Data from 5 independent experiments are presented as median ± interquartile range. Statistical significance (**P < 0.01) was determined by two-tailed Mann-Whitney test.

Figure S3.

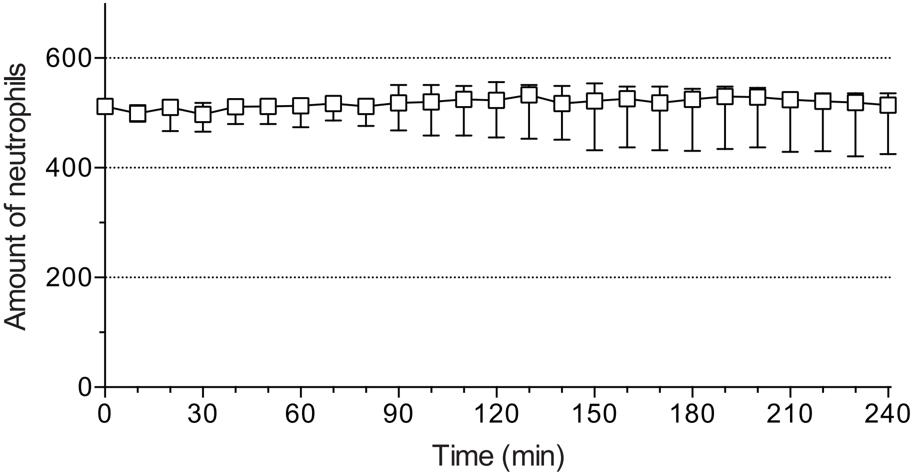

Figure S3. The amount of neutrophils in the field of view over time. Neutrophils from healthy donors were left unstimulated and the amount of neutrophils in the field of view was determined. The amount of neutrophils remains constant over time. Data from 4 independent experiments are presented as median ± interquartile range.

Figure S4.

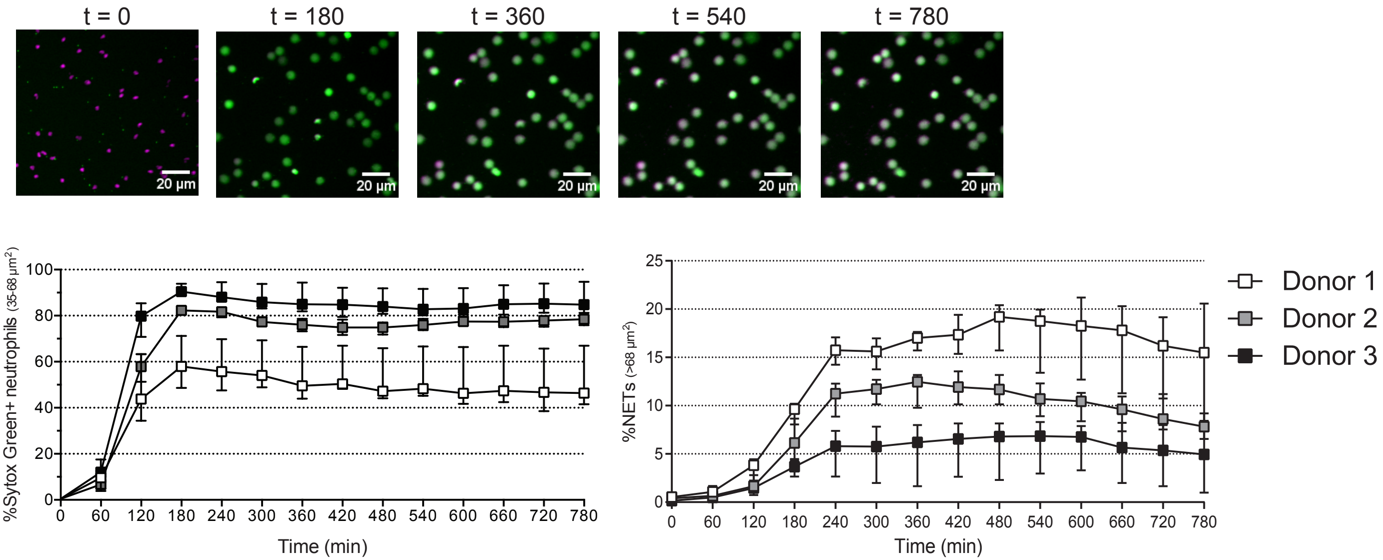

Figure S4. Sytox Green+ neutrophils do not release NETs after prolonged incubation. Neutrophils from healthy donors were stimulated with opsonized *S. aureus* and monitored over time for 13 hours. Quantification of Sytox Green+ neutrophils and NETs of 3 independent experiments performed with 3 donors. The data are presented as median ± interquartile range.

Figure S5.

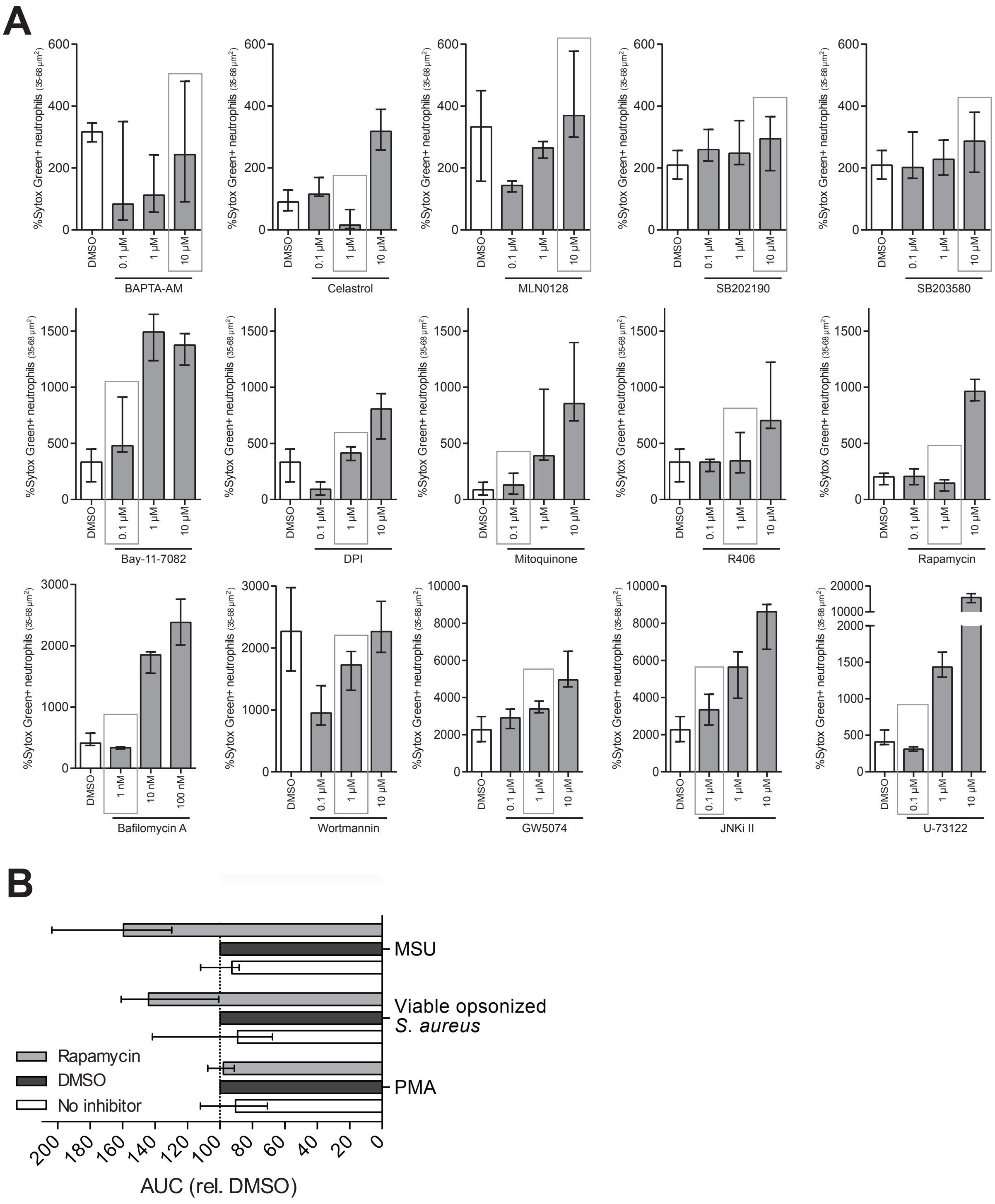
A
%Sytox Green+ neutrophils (35-68 μm²)
DMSO
0.1 μM
1 μM
10 μM
1 nM
10 nM
100 nM
BAPTA-AM
Celastrol
MLN0128
SB202190
SB203580
Bay-11-7082
DPI
Mitoquinone
R406
Rapamycin
Bafilomycin A
Wortmannin
GW5074
JNKi II
U-73122
B
MSU
Viable opsonized
S. aureus
PMA
Rapamycin
DMSO
No inhibitor
AUC (rel. DMSO)

C

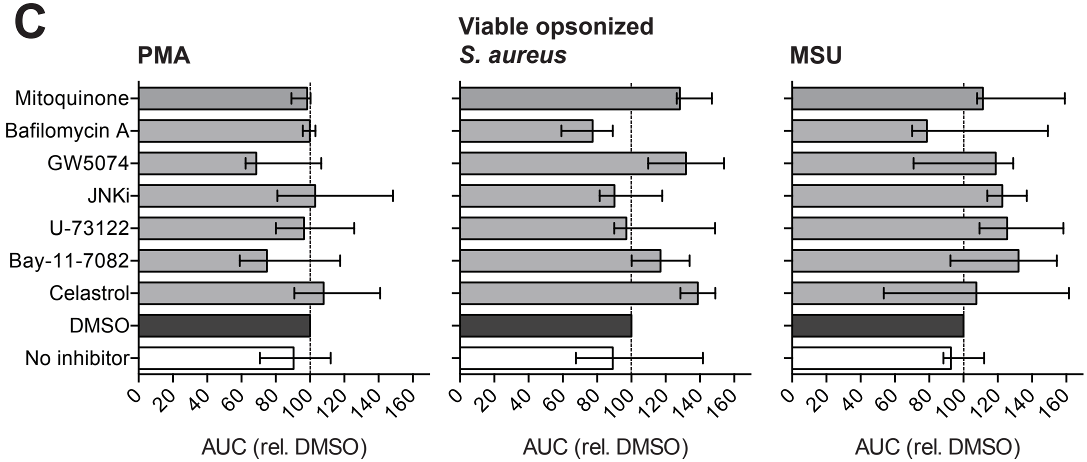

Figure S5. Study of underlying signalling of NET release. (A) A pilot study was performed to determine the optimal concentration of the chemical inhibitors. Neutrophils from healthy donors were incubated with different inhibitor concentration and the concentration that did not show any Sytox Green+ neutrophils (dying cells) was selected (indicated in the gray box). AUC of NET quantification of 2 independent experiments are presented as median ± interquartile range. Neutrophil from healthy donors were pretreated with (B) Rapamycin (mTORC1 inhibitor) or (C) Celastrol (NF-κB inhibitor), Bay-11-7082 (IκB phosphorylation inhibitor), GW5074 (cRaf1 kinase inhibitor), U-73122 (PLC inhibitor), Bafilomycin A (vacuolar-type H+-ATPase inhibitor), JNKi (JNK inhibitor) and Mytoquinone (mitochondrial ROS inhibitor) before stimulation with MSU, PMA and viable opsonized *S. aureus*. AUC of NET quantification of 3 independent experiments are presented as median ± interquartile range.

Figure S6.

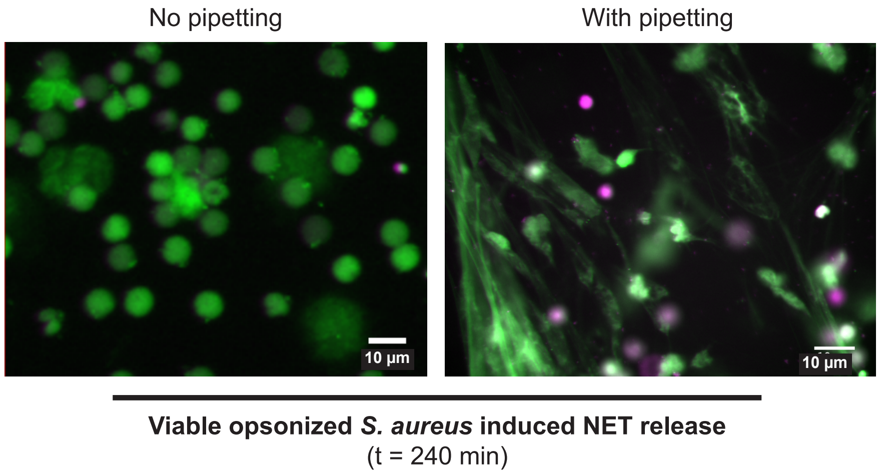

Figure S6. Pipetting artifacts. Neutrophils from a healthy donor were stimulated with opsonized *S. aureus* for 240 min. Merge images (Hoechst 33342 in magenta and Sytox Green in green) show the morphology of NETs with and without pipetting after NET release.
